# Minimum information about a single amplified genome (MISAG) and a metagenome-assembled genome (MIMAG) of bacteria and archaea

**DOI:** 10.1038/nbt.3893

**Published:** 2017-08-01

**Authors:** Robert M Bowers, Nikos C Kyrpides, Ramunas Stepanauskas, Miranda Harmon-Smith, Devin Doud, T B K Reddy, Frederik Schulz, Jessica Jarett, Adam R Rivers, Emiley A Eloe-Fadrosh, Susannah G Tringe, Natalia N Ivanova, Alex Copeland, Alicia Clum, Eric D Becraft, Rex R Malmstrom, Bruce Birren, Mircea Podar, Peer Bork, George M Weinstock, George M Garrity, Jeremy A Dodsworth, Shibu Yooseph, Granger Sutton, Frank O Glöckner, Jack A Gilbert, William C Nelson, Steven J Hallam, Sean P Jungbluth, Thijs J G Ettema, Scott Tighe, Konstantinos T Konstantinidis, Wen-Tso Liu, Brett J Baker, Thomas Rattei, Jonathan A Eisen, Brian Hedlund, Katherine D McMahon, Noah Fierer, Rob Knight, Rob Finn, Guy Cochrane, Ilene Karsch-Mizrachi, Gene W Tyson, Christian Rinke, The Genome Standards Consortium, Nikos C Kyrpides, Lynn Schriml, George M Garrity, Philip Hugenholtz, Granger Sutton, Pelin Yilmaz, Folker Meyer, Frank O Glöckner, Jack A Gilbert, Rob Knight, Rob Finn, Guy Cochrane, Ilene Karsch-Mizrachi, Alla Lapidus, Folker Meyer, Pelin Yilmaz, Donovan H Parks, A Murat Eren, Lynn Schriml, Jillian F Banfield, Philip Hugenholtz, Tanja Woyke

**Affiliations:** 1 Department of Energy Joint Genome Institute, Walnut Creek, California USA; 2 Bigelow Laboratory for Ocean Sciences, East Boothbay, Maine USA; 3 United States Department of Agriculture, Agricultural Research Service, Genomics and Bioinformatics Research Unit, Gainesville, Florida USA; 4 School of Natural Sciences, University of California Merced, Merced, California USA; 5 Broad Institute, Cambridge, Massachusetts USA; 6 Biosciences Division, Oak Ridge National Laboratory, Oakridge, Tennessee USA; 7 Structural and Computational Biology Unit, European Molecular Biology Laboratory, Heidelberg, Germany; 8 The Jackson Laboratory for Genomic Medicine, Farmington, Connecticut USA; 9 Department of Microbiology & Molecular Genetics, Biomedical Physical Sciences, Michigan State University, East Lansing, Michigan USA; 10 Department of Biology, California State University, San Bernardino, California USA; 11 J. Craig Venter Institute, San Diego, California USA; 12 J. Craig Venter Institute, Rockville, Maryland USA; 13 Microbial Genomics and Bioinformatics Research Group, Max Planck Institute for Marine Microbiology, Bremen, Germany; 14 Biosciences Division, Argonne National Laboratory, Argonne, Illinois USA; 15 Department of Surgery, University of Chicago, Chicago, Illinois USA; 16 Biological Sciences Division, Earth and Biological Sciences Directorate, Pacific Northwest National Laboratory, Richland, Washington USA; 17 Department of Microbiology & Immunology, University of British Columbia, Vancouver, British Columbia Canada; 18 Center for Dark Energy Biosphere Investigation, University of Southern California, Los Angeles, California USA; 19 Department of Cell and Molecular Biology, Science for Life Laboratory, Uppsala University, Uppsala, Sweden; 20 Advanced Genomics Lab, University of Vermont Cancer Center, Burlington, Vermont USA; 21 Georgia Institute of Technology, School of Civil and Environmental Engineering, Atlanta, Georgia USA; 22 Department of Civil and Environmental Engineering, University of Illinois at Urbana-Champaign, Urbana, Illinois USA; 23 Department of Marine Science, University of Texas-Austin, Marine Science Institute, Austin, Texas USA; 24 Department of Microbiology and Ecosystem Science, University of Vienna, Vienna, Austria; 25 Genome Center, University of California, Davis, California USA; 26 School of Life Sciences, University of Nevada Las Vegas, Las Vegas, Nevada USA; 27 Nevada Institute of Personalized Medicine, University of Nevada Las Vegas, Las Vegas, Nevada USA; 28 Department of Civil and Environmental Engineering, University of Wisconsin-Madison, Madison, Wisconsin USA; 29 Department of Bacteriology, University of Wisconsin-Madison, Madison, Wisconsin USA; 30 Cooperative Institute for Research in Environmental Sciences, University of Colorado, Boulder, Colorado USA; 31 Department of Ecology and Evolutionary Biology, University of Colorado, Boulder, Colorado USA; 32 and Departments of Pediatrics and Computer Science & Engineering, Center for Microbiome Innovation, University of California San Diego, La Jolla, California USA; 33 European Molecular Biology Laboratory, European Bioinformatics Institute (EMBL-EBI), Welcome Trust Genome Campus, Hinxton, Cambridge UK; 34 National Center for Biotechnology Information, National Library of Medicine, National Institutes of Health, Bethesda, Maryland USA; 35 Australian Centre for Ecogenomics, School of Chemistry and Molecular Biosciences, The University of Queensland, Brisbane, Queensland Australia; 37 Centre for Algorithmic Biotechnology, ITBM, St. Petersburg State University, St. Petersburg, Russia; 38 Department of Medicine, University of Chicago, Chicago, Illinois USA; 41 Marine Biological Laboratory, Woods Hole, Massachusetts USA; 39 National Cancer Institute, Frederick, Maryland USA; 40 Department of Earth and Planetary Science, University of California, Berkeley, California USA

**Keywords:** Genetic databases, Environmental microbiology

## Abstract

We present two standards developed by the Genomic Standards Consortium (GSC) for reporting bacterial and archaeal genome sequences. Both are extensions of the Minimum Information about Any (x) Sequence (MIxS). The standards are the Minimum Information about a Single Amplified Genome (MISAG) and the Minimum Information about a Metagenome-Assembled Genome (MIMAG), including, but not limited to, assembly quality, and estimates of genome completeness and contamination. These standards can be used in combination with other GSC checklists, including the Minimum Information about a Genome Sequence (MIGS), Minimum Information about a Metagenomic Sequence (MIMS), and Minimum Information about a Marker Gene Sequence (MIMARKS). Community-wide adoption of MISAG and MIMAG will facilitate more robust comparative genomic analyses of bacterial and archaeal diversity.

**Supplementary information:**

The online version of this article (doi:10.1038/nbt.3893) contains supplementary material, which is available to authorized users.

## Abstract (web summary)

Standards for sequencing the microbial 'uncultivated majority', namely bacterial and archaeal single-cell genome sequences, and genome sequences from metagenomic datasets, are proposed.

**Supplementary information:**

The online version of this article (doi:10.1038/nbt.3893) contains supplementary material, which is available to authorized users.

## Main

The term “uncultivated majority” was coined to denote the fraction of microbes that have not yet been isolated and grown in axenic culture^1,2^. This diversity was originally identified by sequencing phylogenetically relevant genes, notably the 16S ribosomal RNA gene, and more recently characterized by shotgun metagenomics^3,4^ and single-cell genomics^5,6^. Large-scale sequencing efforts that accelerated discovery of this diversity, such as the Human Microbiome Project^7^, the Earth Microbiome Project^8^, and the Genomic Encyclopedia of Bacteria and Archaea^9^ have improved our understanding of microbial diversity and function as it relates to human health, biogeochemical cycling, and the evolutionary relationships that structure the tree of life.

**Figure 1:**
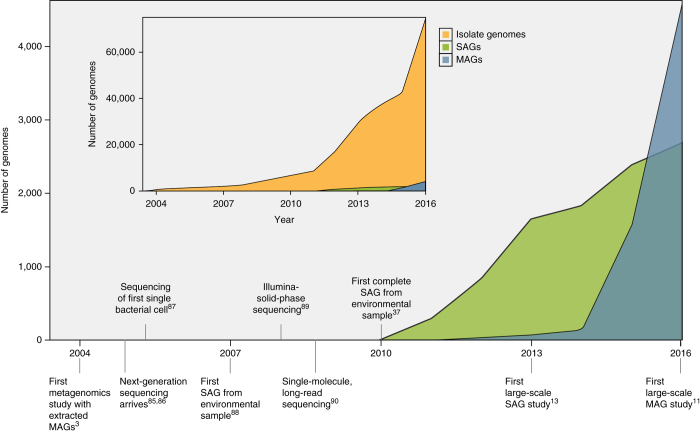
Sequencing of bacterial and archaeal genomes^3,11,13,37,85,86,87,88,89,90^. Increase in the number of SAGs and MAGs over time. Inset displays the number of isolate genomes over time for comparison. Data for figure were taken from IMG/GOLD^14^ in January 2017.

With advances in sequencing technologies, throughput, and bioinformatics approaches, tens to hundreds and even thousands of microbial genomes can be retrieved from complex samples without cultivation of any of the community members^10,11,12,13^. There are 2,866 single-cell genomes and 4,622 genomes reconstructed from metagenomes, which are already registered in the Genomes OnLine Database (GOLD)^14^ (Fig. 1). These numbers are increasing rapidly and will soon outpace the rate of sequencing of cultivated microbial isolate genomes^10^.

As this field matures, it is crucial to define minimum standards for the generation, deposition, and publication of genomes derived from uncultivated bacteria and archaea and to capture the appropriate metadata in a consistent and standardized manner, in line with previous efforts for cultivated isolate genomes^15,16^ and marker gene surveys^17^.

The GSC (http://gensc.org) maintains up-to-date metadata checklists for the MIxS, encompassing MIGS^15^, MIMS^15^, and MIMARKS^17^. Complementing these standards are the Minimum Information about a Biosynthetic Gene Cluster^18^ and the Minimum Information about Sequence Data and Ecosystem Metadata from the Built Environment^19^. Here, we develop a set of standards that extend the MIxS checklists. Our standards form a set of recommendations for the generation, analysis, and reporting of bacterial and archaeal single amplified genomes (SAGs) and metagenome-assembled genomes (MAGs; Table 1 and Supplementary Table 1). We hope that these standards will promote the collection and reporting of appropriate contextual metadata necessary to support large-scale comparative studies and assist researchers with retrieving genomes of uncultivated microorganisms from, and depositing them to, the international nucleotide sequence databases.

**Table 1:** Genome reporting standards for SAGs and MAGs

Criterion	Description
**Finished (SAG/MAG)**	
Assembly quality^a^	Single contiguous sequence without gaps or ambiguities with a consensus error rate equivalent to Q50 or better
**High-quality draft (SAG/MAG)**	
Assembly quality^a^	Multiple fragments where gaps span repetitive regions. Presence of the 23S, 16S, and 5S rRNA genes and at least 18 tRNAs.
Completion^b^	>90%
Contamination^c^	<5%
**Medium-quality draft (SAG/MAG)**	
Assembly quality^a^	Many fragments with little to no review of assembly other than reporting of standard assembly statistics.
Completion^b^	≥50%
Contamination^c^	<10%
**Low-quality draft (SAG/MAG)**	
Assembly quality^a^	Many fragments with little to no review of assembly other than reporting of standard assembly statistics.
Completion^b^	<50%
Contamination^c^	<10%
This is a compressed set of genome reporting standards for SAGs and MAGs. For a complete list of mandatory and optional standards, see Supplementary Table 1.	

^a^Assembly statistics include but are not limited to: N50, L50, largest contig, number of contigs, assembly size, percentage of reads that map back to the assembly, and number of predicted genes per genome.

^b^Completion: ratio of observed single-copy marker genes to total single-copy marker genes in chosen marker gene set.

^c^Contamination: ratio of observed single-copy marker genes in ≥2 copies to total single-copy marker genes in chosen marker gene set.

Our standards feature mandatory requirements, but are flexible enough to accommodate changes over time. For example, as sequence read lengths increase, new methods for assembly and metagenomic binning will likely be devised, and, consequently, sequence databases will need to be updated with metadata that include different sequencing platforms and analysis pipelines. Additionally, as completely new phylogenetic clades are discovered by sequencing, conserved marker gene sets that are used to estimate genome completeness will need to be updated to place new data in the appropriate context.

**Minimum information about SAGs and MAGs**

**Figure 2:**
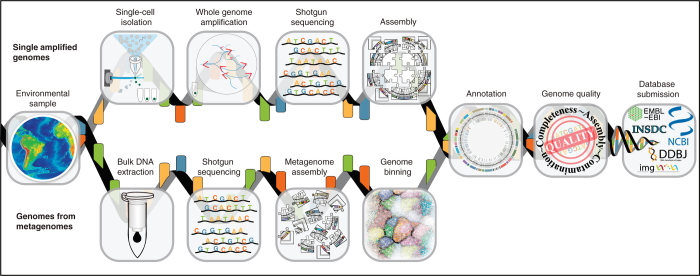
Generation of SAGs and MAGs. Flow diagram outlining the typical pipeline for the production of both SAGs and MAGs.

SAGs are produced by isolating individual cells, amplifying the genome of each cell using whole genome amplification (WGA), and then sequencing the amplified DNA^6,20^. MAGs, on the other hand, are produced using computational binning tools that group assembled contigs into genomes from Gbp-level metagenomic data sets^21,22,23,24^ (Fig. 2 and Supplementary Table 1). Both SAGs and MAGs are often highly fragmented and are sometimes contaminated with non-target sequence. Owing to these challenges, we propose that SAGs and MAGs need to have some shared metadata (Supplementary Table 1). Our standards extend the MIxS checklists by including additional criteria to assess SAG and MAG quality, which will soon become core standards required for submission to suitable databases such as those found at the National Center for Biotechnology Information (NCBI) and the European Molecular Biology Laboratory-European Bioinformatics Institute (EMBL-EBI; Hinxton, UK), the DNA Database of Japan (DDBJ) and GOLD.

**Single amplified genomes.** Sequencing of genomes from single cells requires specialized instrumentation, such as flow cytometry, microfluidics, or micromanipulators for single-cell isolation, and cleanrooms for downstream handling (Supplementary Table 1)^20,25,26,27^. Given the extremely low yields of genomic DNA from a single microbial cell (∼1–6 fg)^28^, DNA must be amplified after cell lysis to generate the quantities required for currently available sequencing technologies. The most commonly used method for WGA is multiple displacement amplification (MDA)^29^, which relies on the highly processive Phi^29^ DNA polymerase^30^. MDA yields significant coverage biases^31^, alters GC profiles^32^, and produces chimeric molecules during the amplification reaction^33^, but remains the primary method for WGA of single cells. Recent advances in assembly algorithms, including single-cell-specific assemblers that use multiple coverage cutoffs (e.g., SPAdes (St. Petersburg Genome Assembler)^34^ and IDBA-UD (Iterative De Bruijn Graph *De Novo* Assembler for Short Reads Sequencing Data with Highly Uneven Sequencing Depth)^35^), along with a number of publicly available k-mer coverage normalization tools^36,37^, have provided researchers with some tools to tackle the chimeric and biased nature of single-cell sequence data.

Because most bacterial and archaeal cells contain a single or very few genome copies, introducing even trace amounts of contaminant DNA during cell sorting, lysis, or WGA can severely affect downstream SAG data quality. Contamination can originate from multiple sources, including the samples themselves, the laboratory environment, reagents supplied by vendors^25,27,38^, and library poolmates when multiplexing samples for sequencing. Furthermore, the lack of corresponding laboratory cultures from which genomes could be resequenced and validated using alternative methods presents a fundamental challenge in evaluating the accuracy of SAG assemblies. One way to address this challenge is to benchmark the entire workflow by using mock communities of well-characterized laboratory strains. Comparing the benchmark assemblies to genomes included in a mock sample could provide an estimate of probable errors in novel SAGs from uncultivated microbes. Published benchmark studies have revealed infrequent mismatches (∼9/100 kb), indels (∼2/100 kb), and misassemblies (∼1/Mb) in single-cell genomes^39^.

The ideal scenario is to produce contaminant-free SAGs^20^, but as this is not always possible, tools that can detect and eliminate potential contamination at the read and contig (assembly) levels have been developed. Tools for read decontamination, including DeconSeq^36^, and modules from the BBtools package, such as bbduk.sh (https://sourceforge.net/projects/bbmap/) remove contaminant sequences from query genomes based on user-defined contaminant databases. Quality assurance and/or decontamination of assembled SAGs has primarily been a semi-manual process that scrutinizes a variety of genomic attributes, such as non-target 16S rRNA genes, abnormal k-mer frequencies, and/or variable GC content^37^. However, more automated tools that identify contaminant contigs in genomic data sets have recently become available, including Anvi'o (Analysis and Visualization Platform for 'Omics Data)^40^, CheckM^41^, ProDeGe (Protocol for Fully Automated Decontamination of Genomes)^42^, and acdc (Automated Contamination Detection and Confidence Estimation)^43^. Taxonomic assignment of SAGs is generally based on marker gene phylogenies or the 16S rRNA gene sequence^20^.

There are no definitions and/or guidelines for either the assembly, quality control, and classification of SAGs, or the criteria to assess the final SAG assembly and how to associate the metadata with the assembled genomes.

**Metagenome-assembled genomes.** Assembly of microbial genomes from metagenomic sequence reads was pioneered in 2004 by Tyson *et al*.3 by extracting near-complete genomes from a metagenome of an acid mine drainage community that contained only a few bacterial and archaeal taxa. Although assembly of complete microbial genomes was initially restricted to environmental samples with exceptionally low microbial diversity^3,44,45^, increasing sequencing throughput, read lengths, and improved assembly and binning algorithms have enabled genome-resolved metagenomics to be carried out for communities with high diversity^10,11,21,46^. To generate a genome, metagenomic sequence reads are assembled into contigs using metagenome-specific algorithms^35,47,48,49^ and contigs are grouped, and these groups are then assigned to discrete population bins^3,4,50^.

Criteria used by metagenomic binning software include nucleotide sequence signatures (e.g., GC content and/or tetra-nucleotide frequency), marker gene phylogenies, depth of DNA sequence coverage, and abundance patterns across samples^51^. If these features are combined, bins of high quality can be produced^52^. Metagenomic binning has proven powerful for the extraction of genomes of rare community members (<1%). For example, differential coverage binning has been used recently to extract near-complete genomes of the low-abundance candidate phylum TM7 (Saccharibacteria) from wastewater bioreactor samples^21^. Other approaches have used differential coverage binning to identify species and strains during a time course of gut microbiome development in a newborn infant from 15 to 24 days after delivery^53^. In a more recent study, >2,500 MAGs were extracted from below-ground sediment and aquifer samples, taking advantage of nucleotide composition signatures, abundance of organisms across samples, and the taxonomic association of metabolic genes^10^. Tools are available that take advantage of multi-parameter binning, such as GroopM^54^, MaxBin^55^, MetaBAT (Metagenome Binning with Abundance and Tetranucleotide Frequencies)^56^, CONCOCT^57^, and MetaWatt^58^. Taxonomic identity of the bins can be assigned by marker gene phylogeny or using the 16S rRNA gene sequence^11^.

There are no strict definitions and/or guidelines for how to assemble and bin genomes from metagenomes, which parameters to use, how to taxonomically classify and define the end product, or how to include the metadata with the assembled genomes.

**Developing MISAG and MIMAG checklists**

The three most important criteria for assessing SAG and MAG quality are assembly quality, genome completeness, and a measure of contamination. These criteria are discussed below and their associated standards are summarized in Table 1 (in full in Supplementary Table 1).

For both SAGs and MAGs, assessing assembly quality is non-trivial due to the lack of a 'ground truth'. This is because SAGs and MAGs most often come from organisms that lack a cultivated reference strain. To assist downstream users in the evaluation of assembly quality, we recommend reporting basic assembly statistics from individual SAGs and/or MAGs, including, total assembly size, contig N50/L50, and maximum contig length (Supplementary Table 1). Contigs should not be artificially concatenated before deposition, as the resulting concatenation is not a true representation of the genome. We do not suggest a minimum assembly size, because genomes smaller than 200 kb have been found among symbiotic bacteria^59,60,61^. Lastly, the presence and completeness of the complement of encoded rRNAs and tRNAs should be used as an additional metric for assembly quality (Table 1). Because these draft genome sequences are not manually curated, the assembly quality standards of Chain *et al*.^16^ are not well-suited to SAGs and MAGs. However, in some cases, MAGs are manually curated, sometimes to completion, in which case the standards laid out in Chain *et al*.^16^ would be applicable.

The fraction of the genome captured from a SAG and MAG is another important metric because the level of completeness could dictate whether a publicly available genome is suitable for a specific downstream analysis. For example, complete genomes are preferable for pangenome analyses and genetic linkage studies^62^, whereas partial genomes may be suitable for fragment recruitment analyses^26,63^, metabolic predictions^11^, and phylogenetic reconstruction of individual proteins^64^. There are no established standards for estimating SAG and MAG completeness. The ideal approach might be to map a SAG or MAG to a closely related reference genome sequence. However, this is often not possible given the lack of suitable references for many microbial lineages and high levels of strain heterogeneity^65,66,67^. Alternatively, researchers have relied on the presence of 'universal' marker genes to estimate completeness. An appropriate marker gene should be present in genomes of nearly all taxa, as a single copy, and not subject to horizontal gene transfer. Although a discussion of approaches to identify such gene sets is beyond the scope of this manuscript, several gene sets have been identified and validated, some of which span both archaeal and bacterial domains^68,69,70,71^, whereas others are specific to archaeal^13^ or bacterial^13,72,73^ genomes. Many of these gene sets are now included in MAG and SAG quality assessment software, such as CheckM^41^, Anvi'o^40^, mOTU (Metagenomic Operational Taxonomic Units)^74^, and BUSCO (Benchmarking Universal Single-Copy Orthologs)^71^. Because different gene sets can produce different completeness estimates, the set chosen should be based on an established collection, previously validated and published in the literature (any of the above-mentioned sets would be sufficient), or the process of gene selection should be documented. Ribosomal proteins are included in gene sets, but because these genes tend to cluster unevenly across the genome, completeness estimates can be skewed^75^. To account for this bias, many of the marker sets include housekeeping genes involved in replication and transcription. The CheckM tool takes gene selection a step further by inferring lineage-specific genes based on the position of a query genome in a reference tree using a reduced set of multi-domain markers^41^. We recommend that MISAG- and MIMAG-compliant submissions use any of the previously mentioned single-copy marker gene sets, or follow a strategy similar to the one used by CheckM to identify gene sets; documentation of the selection process is considered mandatory. Gene sets must also be versioned, so that metadata can clearly indicate the procedure used.

Finally, the fraction of a SAG or MAG that may contain contaminating sequences should be reported. There are many highly recommended tools and techniques that can reduce or remove contaminating DNA in a genome before database submission (see sections on 'Single amplified genomes' and 'Metagenome-assembled genomes', and Supplementary Table 1 under 'decontamination software'). These approaches typically calculate the fraction of single-copy genes used in completeness estimates that are present more than once in a genome^21,41,76,77^, although contamination can be overestimated when a gene is artificially split at contig ends and scaffolding points. Tools, such as Anvi'o^40^ and CheckM^41^, can iteratively scan genomes for contamination to identify contaminant sequences. Both of these tools estimate contamination and provide several functions to enable users to remove contaminating sequences. Finally, we encourage researchers to carry out manual quality control based on nucleotide composition and BLAST-based analyses to identify suspicious contigs. Manual screening can be time consuming, although tools like Anvi'o have enabled interactive decontamination based on relevant parameters, such as GC content, tetranucleotide frequency, coverage, taxonomy, and combinations of these parameters^78^.

**Mandatory standard metrics**

We suggest that assembly statistics and estimates of genome completeness and contamination for SAGs and MAGs be mandatory metrics for both reporting in publications and deposition in public databases. Using these simple standards, we recommend that each genome be classified as: finished, high-quality draft, medium-quality draft, or low-quality draft (Table 1 and Supplementary Table 1). Mandatory standards are listed in Table 1, with the full set of standards (including optional and context-dependent) standards listed in Supplementary Table 1. A 'finished' category is reserved for genomes that can be assembled with extensive manual review and editing, into a single, validated, contiguous sequence per replicon, without gaps or ambiguities, having a consensus error rate equivalent to Q50 or better^16^. This category is reserved for only the highest quality manually curated SAGs and MAGs, and several finished genomes have been produced using these technologies^10,11,21,37,79,80,81,82^. For MAGs, genomes in this category are to be considered population genomes. 'High-quality draft' will indicate that a SAG or MAG is >90% complete with less than 5% contamination. Genomes in this category should also encode the 23S, 16S, and 5S rRNA genes, and tRNAs for at least 18 of the 20 possible amino acids, as even the reduced genomes of bacterial symbionts typically harbor the full complement of tRNAs^83,84^. 'Medium-quality draft' SAGs and MAGs are those genomes with completeness estimates of ≥50% and less than 10% contamination (Table 1 and Supplementary Table 1). All other SAGs and MAGs (<50% complete with <10% contamination) should be reported as 'low-quality drafts' (Table 1 and Supplementary Table 1).

All SAG and MAG public database submissions should include, at the very least, the metadata listed as mandatory in Supplementary Table 1. Additional standards include information about the assembly and binning software used and tools to taxonomically identify the genome. Owing to the many experimental and computational challenges associated with the generation of SAGs and MAGs, these minimum standards should be rigorously enforced in future genome submissions.

**Conclusions**

The GSC standards outlined here are a necessary extension of the MIxS standards, owing to the vast difference between generating genome sequences from cultivated versus uncultivated bacteria and archaea. These recommendations will serve to promote discussion and to generate feedback and subsequent improvements, which is especially relevant in the rapidly changing landscape of genomics technologies. These standards will be incorporated into the current GSC checklists and will complement the MIGS, MIMS, and MIMARKS checklists.

**Publisher's note**

Springer Nature remains neutral with regard to jurisdictional claims in published maps and institutional affiliations.

## Supplementary information

Supplementary Tables Supplementary Table 1 (XLSX 19 kb)
